# 

**Published:** 1877-01-20

**Authors:** 


					﻿K
K
Ou
pH
5
o
a
I
■
p
; o
! t*
I H
■■ <
i Qj
P
*"7
X
! E
H
, fe
! O
OD
■5
P
Q
1 a
1 M
E-
Eh
I
I a
QQ-
! B
' «
! Ph
i K
I CO
I <
I a
i a
o-
VOL. VII.	JANUARY 20, 1877.	No. K
the
SOUTHERN MEDICAL RECORD.
A MONTHLY JOURNAL OF PRACTICAL MEDICINE.
“ QU1CQUJD PR2EC1 VIES ESTO BREVIS.”
$
OQ :
hs
i
g
g
O ,
I
3D |
>	I
I
k>
Q i
o ,:
>	i
W 1
t> I
M I
3D I
O I
i-3
JAS. P. HARRISON & CO., Printers.
ATLANTA, GEORGIA.
TERMS: $2.00 per Annum in Advance—Club Rates, 5 Copies for $8.00.
				

